# Nano Chromium Picolinate Improves Gene Expression Associated with Insulin Signaling in Porcine Skeletal Muscle and Adipose Tissue

**DOI:** 10.3390/ani10091685

**Published:** 2020-09-18

**Authors:** Alex T. Hung, Brian J. Leury, Matthew A. Sabin, Fahri Fahri, Kristy DiGiacomo, Tu-Fa Lien, Frank R. Dunshea

**Affiliations:** 1 Faculty of Veterinary and Agricultural Sciences, The University of Melbourne, Parkville, VIC 3010, Australia; alex.hung@c-pharmachem.com.sg (A.T.H.); brianjl@unimelb.edu.au (B.J.L.); fahri.fahri@dpi.nsw.gov.au (F.F.); kristyd@unimelb.edu.au (K.D.); 2 Murdoch Children’s Research Institute, The University of Melbourne, Parkville, VIC 3010, Australia; matthew.sabin@mcri.edu.au; 3 Department of Animal Science, National Chiayi University, Chiayi 600, Taiwan; tflien@mail.ncyu.edu.tw; 4 Faculty of Biological Sciences, The University of Leeds, Leeds LS2 9JT, UK

**Keywords:** chromium, nanotechnology, insulin sensitivity, cytokine

## Abstract

**Simple Summary:**

Dietary chromium has been shown to reduce fat deposition and improve insulin action whereas dietary fat can increase fat deposition and cause insulin resistance. This study found that dietary nanoparticles of chromium picolinate, an organic form of chromium, caused changes in the genes involved in insulin action in both muscle and fat tissue that indicated improved insulin action. Conversely, a moderate increase in dietary fat caused changes consistent with increased fat deposition and reduced insulin action. In conclusion, nanoparticles of chromium picolinate offer a means of supplementing pigs diets to improve growth performance and carcass composition.

**Abstract:**

The aim of this study was to investigate the interactive effects of dietary nano chromium picolinate (nCrPic) and dietary fat on genes involved in insulin signaling in skeletal muscle and subcutaneous adipose tissue of pigs. Forty-eight gilts were stratified on body weight into four blocks of four pens of three pigs and then within each block each pen was randomly allocated to four treatment groups in a 2 × 2 factorial design. The respective factors were dietary fat (22 or 57 g/kg) and dietary nCrPic (0 or 400 ppb nCrPic) fed for six weeks. Skeletal muscle samples were collected from the *Longissimus thoracis* and subcutaneous adipose tissue collected from above this muscle. Dietary nCrPic increased adiponectin, uncoupling protein 3 (UCP3) and serine/threonine protein kinase (AKT) mRNA expression, whereas dietary fat decreased adiponectin and increased leptin, tumor necrosis factor-*α* (TNF-*α*), peroxisome proliferator-activated receptors *γ* (PPAR*γ*) and CCAAT/enhancer-binding protein *α* (C/EBP*α*) mRNA expression in adipose tissue. In skeletal muscle, dietary nCrPic increased phosphatidylinositol 3 kinase (PI3K), AKT, UCP3 and interleukin-15 (IL-15), as well as decreased suppressor of cytokine signaling 3 (SOCS3) mRNA expression. The improvement in insulin signaling and muscle mass and the reduction in carcass fatness by dietary nCrPic may be via decreased SOCS3 and increased UCP3 and IL-15 in skeletal muscle and increased adiponectin in subcutaneous adipose tissue.

## 1. Introduction

The rapid rise in obesity is a critically important health issue worldwide. Obesity is associated with a number of health problems including the development of insulin resistance. Obesity is characterized by increased storage of fatty acids in an expanded adipose tissue mass and is closely associated with the development of insulin resistance in peripheral tissues such as skeletal muscle and adipose tissue [1]. High dietary fat intake has been implicated as a major cause of insulin resistance with several studies demonstrating an association between high-fat feeding and insulin resistance, suggesting a causal role of dietary fat in the pathophysiology of type 2 diabetes [2,3,4].

Although the molecular mechanisms leading to insulin resistance remain elusive, emerging evidence suggests that a high fat mass or the rate of fat deposition is strongly correlated with insulin resistance [5,6,7]. The transcriptional factors peroxisome proliferator-activated receptors (PPARs), C/EPB and sterol regulatory element-binding proteins (SREBPs) play important roles in adipogenesis [8]. For example, the expression of C/EBPs has been shown to induce the expression of PPAR*γ* during an early stage of adipocyte differentiation in vitro [9]. Expression of PPAR*γ* in adipose tissue promotes the differentiation of preadipocytes and regulates some fat cell-specific genes expression [10]. SREBPs modulate lipogenesis and cholesterol homeostasis and SREBP2 overexpression increases fatty acid syntheses (FAS) gene expression [11]. 

Adipose tissue is now recognized as an endocrine organ that contributes to insulin resistance [12]. In the past decade, a large number of endocrine and inflammatory pathways have been shown to be dysregulated in obesity [5]. Adipokines produced by adipose tissue have been identified as potential contributors to insulin resistance [12]. The representative adipokines produced by adipose tissue include leptin, adiponectin and TNF-*α*. Dysregulated production of these adipokines is involved in the pathogenesis of the obesity-associated metabolic syndrome. For example, overexpression of TNF-α from accumulated fat contributes to the development of insulin resistance in obesity [13]. In contrast, adiponectin is down-regulated in obesity and improves insulin sensitivity [14]. Leptin has been shown to result in increased insulin sensitivity via increased fatty acid *β*-oxidation. However, high-fat diets or development of obesity may lead to leptin resistance in skeletal muscle [15,16,17]. 

Chromium (Cr) is an essential mineral element in both humans and animals [18,19] with trivalent Cr being the most stable form occurring in nature. Cr functions as a cofactor for the hormone insulin and enhances the ability of insulin to regulate glucose, protein and fat metabolism [20]. A dietary deficiency of Cr is believed to be positively associated with the risk of diabetes [21] and obesity [22]. Accordingly, several human and animal studies have demonstrated that exogenous Cr administration improves glucose tolerance and insulin sensitivity in type 2 diabetes and obesity [23,24,25]. The interest in Cr supplementation during the finisher phase of pig production is primarily for its potential impact to improve body composition. However, the effect of Cr on body composition of finisher pigs is inconsistent. Some of the variation in response to Cr may be related to the low absorption and availability of Cr, and there is potential to improve this by reducing the particle size of Cr.

The present study evaluated the effect of dietary nano chromium picolinate (nCrPic) and fat on adipogenesis and insulin signaling using the pig as a biomedical model. Furthermore, in an attempt to understand the potential molecular mechanisms involved, the effect of dietary nCrPic and fat on genes associated with adipokines and insulin resistance was investigated.

## 2. Materials and Methods

The procedures used in the experiment were approved by The University of Melbourne, School of Land and Environment Animal Ethics Committee (permit number: Ethics ID: 0810919.1).

### 2.1. Animals and Treatments

**Figure 1:**
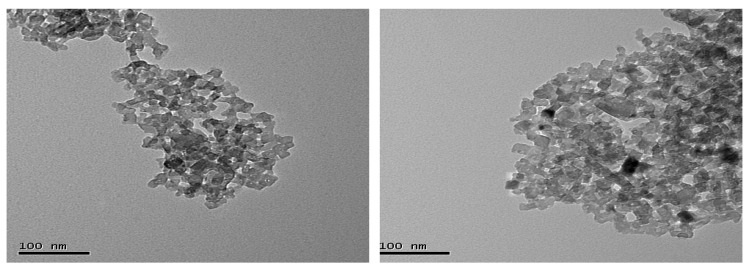
Transmission electron microscopic images of nano CrPic nanoparticles.

The tissue samples used for quantifying mRNA expression in this investigation were obtained from a subset of animals used in a previous experiment [26]. In brief, forty-eight Large white and Landrace cross breed finishing gilts (PrimeGro™ Genetics, Rivalea Pty Ltd. Corowa, NSW, Australia) with and initial weight 52.2 ± 7.3 kg were stratified on weight into four blocks of four pens of three pigs and then within each block each pen was randomly allocated to four treatment groups in a 2 × 2 factorial design. The respective factors were dietary fat (22 or 57 g/kg) and dietary Cr (0 or 400 ppb nCrPic) fed for six weeks. The iso-nitrogenous (160 g/kg crude protein and 9 g/kg total lysine) and iso-caloric (13.8 megajouledigestible energy/kg) diets were based on wheat and wheat byproducts, soybean meal and meat meal with additional fat in the high-fat diet provided by tallow. The nCrPic particles were prepared as described previously [26]. Briefly, the raw CrPic material was ground and then passed through an appropriate-sized end-plate sieve, the particle sizes was 49.7 ± 12.37 nm (mean ± s.d.). The microstructure image of nCrPic is shown in Figure 1. The microstructure of the nCrPic was examined by a transmission electronic microscope (TEM) (Hitachi, H-7100, Japan) equipped with a link energy dispersive spectroscopy system and using a bean energy of 300 KeV. For the TEM study, the washed powder was dispersed in methanol using an ultrasonic bath, and a drop of suspension was placed on a copper grid coated with holey carbon film.

### 2.2. Blood Sampling and Plasma Analyses

Blood samples from the median weight pig in each pen were collected via venepuncture from the anterior vena cava at the end of the experiment and after a 16 h fast. After collection of blood, the samples were placed on ice for 1 h, and then centrifuged for 15 min at 1500 *g*. Plasma was collected and frozen (20 °C) until subsequent analysis for glucose and insulin concentrations to determine the degree of insulin resistance using the homeostatic model assessment (HOMA) method [27]. Plasma insulin concentrations were determined by radioimmunoassay (Millipore, Billerica, MA, USA) while plasma glucose was determined by an enzymatic colorimetric procedure (Thermo Fisher Scientific, Waltham, MA, USA).

### 2.3. Subcutaneous Adipose and Skeletal Muscle Biopsies Collection

All pigs were slaughtered commercially after being fed dietary treatments for six weeks (mean body weight 91.5 ± 10.3 kg). After slaughter, all carcasses were ultrasonically scanned (Pork Scan, Brisbane, Qld, Australia) at the P2 site (65 mm from midline over the last rib) for determination of backfat and muscle depth at the P2 site. Subcutaneous adipose tissue sample from above the *Longissmus thoracis* and skeletal muscle tissue samples from *Longissmus thoracis* were collected from the median weight pig in each pen within 25 min post-slaughter and frozen in liquid nitrogen before preservation at −80 °C for batched gene expression studies.

### 2.4. RNA Extraction and Quantification

Standard techniques were used. Briefly, the total RNA extraction was undertaken using the PureLink™ Micro-to-Midi Total RNA Purification System (Invitrogen, Carlsbad, CA, USA). The PureLink™DNase kit (Invitrogen) was then used to remove genomic DNA. The integrity of RNA was assessed via agarose gel electrophoresis via the Experion automated electrophoresis station and the Experion StdSens Analysis kit according to the manufacturer’s instructions (Bio-Rad Laboratories Inc., Hercules, CA, USA), and total RNA was quantified using a spectrophotometer (Nanodrop ND-1000, Thermo Fisher Scientific, Newark, DE, Waltham, MA, USA) with readings taken at 260 and 280 nm.

### 2.5. Reverse Transcriptase PCR and Real-Time Quantitative-PCR (Q-PCR)

Primer optimum annealing conditions were determined and are given in detail in the Table 1. Extracted RNA was transcribed to complimentary DNA (cDNA) using the SuperScript III First-Strand Synthesis System for RT-PCR (Invitrogen, USA) according to the manufacturer’s instructions. A LightCycler 480 II (Roche Diagnostics) was used for Q-PCR analysis. Each Q-PCR reaction mix consisted of 4 μL of SYBR Green 1 Master (Roche Diagnostics, Basel, Switzerland), 1 μL of forward primer, 1 μL of reverse primer, 1 μL of cDNA, and 3 μL of RNase free water. The following thermal cycling protocol was followed: 1 cycle of pre-denaturation (95 °C for 10 min), followed by 40 cycles of amplification (95 °C for 10 s, 57–63 °C for 10 s (dependent on primer), 72 °C for 10 s), 1 cycle of melting (95 °C for 5 s, 65 °C was then used to remove genomic DNA for 1 min, 97 °C for continuous analysis), and cooling (40 °C for 30 s). Changes in gene expression were calculated as 2^−^^ΔCt^, where C_t_ represents the cycle in which fluorescence threshold is reached and

ΔCt = C_t target gene_ − C_t housekeeping gene_ (1)

with *β*-actin utilized as a standard housekeeping gene.

### 2.6. Statistical Analyses

All gene expression data were analysed as the threshold cycle (cT) relative to that of the housekeeping gene *β*-actin (∆CT) and assessed for main and interactive effects of dietary fat and nCrPic by ANOVA and within a tissue by multiple ANOVA (MANOVA) that included all genes analysed within each tissue. A difference in ∆CT of −1.0 is associated with a doubling (200%) and + 1.0 a halving (50%) of expression and for ease of presentation data are presented as % relative to expression in tissue from gilts fed the control diet without supplemental fat or nCrPic. This form of presentation does not allow for the presentation of error bars although error terms can be found in the supplemental tables. Data are presented graphically in the manuscript and tabulated in supplementary Table S1 (adipose tissue) and Table S2 (skeletal muscle). In addition, correlations have been conducted between all mRNA expressions (as ΔCt) as well as with the phenotypic measures of HOMA, back fat depth, loin muscle depth and carcass weight. These correlation coefficients are to be found in a supplemental table (Table S3). All statistical analysis were conducted using GENSTAT (16th edition, Hemel Hempstead, UK). A value of *p* < 0.05 was used to indicate statistical significance and *p* < 0.10 to indicate a trend. 

## 3. Results

### 3.1. Carcass Composition and Plasma Homeostatic Model Assessment of Insulin Resistance

The growth performance and carcass characteristics have been reported elsewhere [26] but in order to relate the gene expression data to the physiological state of the pigs, some of the data are presented in brief here. Dietary nCrPic increased the size of the *Longissmus thoracis* muscle as indicated by muscle depth (49.0 vs. 53.6 mm, standard error of difference (s.e.d.) = 1.13 mm, *p* = 0.004) and decreased the subcutaneous fat thickness (8.2 vs. 7.4 mm, s.e.d. = 0.24 mm, *p* = 0.018). While there were no main effects of dietary fat there were interactions between dietary nCrPic and fat for muscle depth (*p* = 0.019) and fat depth (*p* = 0.042) such that the effects of nCrPic were most pronounced in pigs fed the high-fat diet. Insulin resistance as determined using the homeostatic model assessment was decreased by dietary nCrPic (1.25 vs. 0.66, s.e.d. = 0.147, *p* = 0.009).

### 3.2. Adiponectin, Leptin, TNFα and c-Jun N-Terminal Kinase (JNK1) Gene Expression in Porcine Adipose Tissue

**Figure 2:**
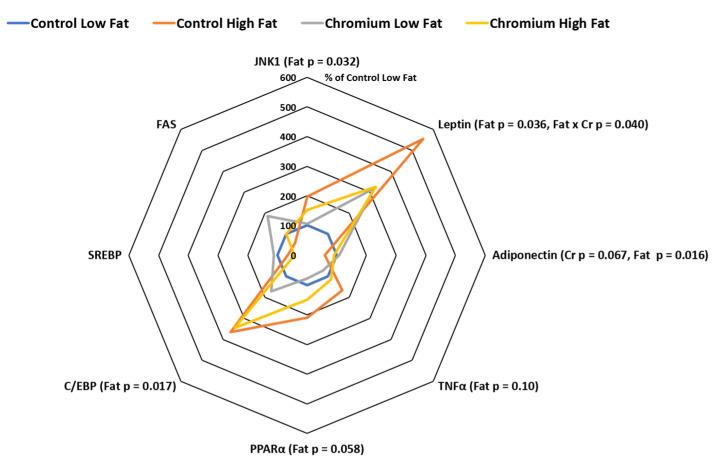
Leptin, adiponectin, tumor necrosis factor *α* (TNF*α*), peroxisome proliferator-activated receptor *γ* (PPAR*γ*), CCAAT enhancer binding protein α (C/EBP*α*), sterol regulatory element-binding protein (SREBP), fatty acid synthase (FAS) and mitogen-activated protein kinase-8 (JNK1) mRNA expression in subcutaneous adipose tissue from gilts fed with nCrPic with or without a high-fat diet. All gene expression data were analysed as the threshold cycle (cT) relative to that of β-actin (∆CT) and assessed for main and interactive effects of dietary fat and nCrPic by ANOVA. A difference in ∆CT of −1.0 is associated with a doubling (200%) and +1.0 a halving (50%) of expression and for ease of presentation data are presented as % relative to expression in tissue from gilts fed the control diet without supplemental fat or nCrPic. This method of presentation prevents the presentation of the error term.

The expression of subcutaneous adipose tissue leptin was doubled by the high-fat diet (relative expression was 210 and 441% for main effects of low- and high-fat diets, respectively, (*p* = 0.036). However, there was also an interaction (*p* = 0.040) between dietary fat and nCrPic such that adipose tissue leptin mRNA expression was increased around 5.5 fold by the high-fat diet alone but not when dietary nCrPic was fed (Figure 2). Similarly, the expression of TNF*α* in adipose tissue tended to be higher in pigs fed the high-fat diet (relative expression was 86 and 141% for main effects of low- and high-fat diets respectively, *p* = 0.10). However, there were no statistical differences in the expression of leptin mRNA (*p* = 0.39) and TNF-*α* mRNA (*p* = 0.23) between pigs fed nCrPic and their controls. The expression of adiponectin in adipose tissue was higher in pigs fed the high-fat diet (relative expression was 105 and 75% for main effects of low- and high-fat diets, respectively, *p* = 0.016) and lower in the nCrPic treatment group (relative expression was 80% and 100% for main effects of control and nCrPic diets, respectively, *p* = 0.067). The expression of mitogen-activated protein kinase-8 JNK1 was increased by the high-fat diet with the relative expression being 175% of the low-fat diet (*p* = 0.032). There was no effect of dietary nCrPic on expression of JNK1.

### 3.3. PPARγ, C/EBPα, SREBP and FAS Gene Expression in Porcine Adipose Tissue

The expression of PPAR*γ* (relative expression was 89%and 180% for main effects of low- and high-fat diets respectively, *p* = 0.058) and C/EBPα (relative expression was 136% and 356% for main effects of low- and high-fat diets respectively, *p* = 0.017) were higher in adipose tissue from pigs fed high-fat diets. On the other hand, there were no main effects of dietary nCrPic on the adipose tissue expression of PPAR*γ*, C/EBP*α*, SREBP and FAS (Figure 2). There were no significant effects of dietary fat or nCrPic on the expression of SREBP and fatty acid synthase in porcine adipose tissue (Figure 2). 

### 3.4. IR, PI3K, AKT, UCP3 and SOCS3 Gene Expression in Porcine Adipose Tissue

**Figure 3:**
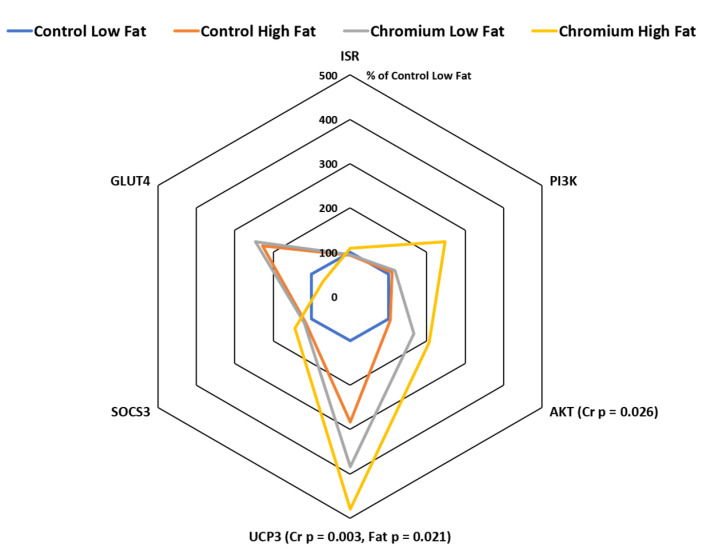
Phosphatidylinositol 3 kinase (PI3K), protein kinase B (AKT), uncoupling protein 3 (UCP3), suppressor of cytokine signaling 3 (SOCS3), glucose transporter 4 (GLUT4) and insulin receptor (IRS) mRNA expression in subcutaneous adipose tissue from gilts fed with nCrPic with or without a high-fat diet. All gene expression data were analysed as the threshold cycle (cT) relative to that of *β*-actin (∆CT) and assessed for main and interactive effects of dietary fat and nCrPic by ANOVA. A difference in ∆CT of −1.0 is associated with a doubling (200%) and +1.0 a halving (50%) of expression and for ease of presentation data are presented as % relative to expression in tissue from gilts fed the control diet without supplemental fat or nCrPic. This method of presentation prevents the presentation of the error term.

The expression of the insulin signaling pathway gene AKT was higher in pigs fed the nCrPic diet (relative expression was 103 and 186% for main effects of control and nCrPic diet, respectively, *p* = 0.026) (Figure 3). However, dietary nCrPic had no effect on adipose tissue IR (*p* = 0.91) and phosphatidylinositol 3 kinase (PI3K) (*p* = 0.59) mRNA expression. The expression of the UCP3 gene in adipose tissue was higher in pigs fed dietary nCrPic (relative expression was 191 and 431% for main effects of control and nCrPic diets, respectively, *p* = 0.003) and lower in pigs fed the high-fat diet (relative expression was 242 and 381% for low- and high-fat diets, respectively, *p* = 0.021).There was no effect of either dietary fat or nCrPic on suppressor of cytokine signaling 3 (SOCS3) or GLUT4 gene expression (Figure 3).

### 3.5. IR, PI3K, AKT, GLUT4 and SOCS3 Gene Expression in Porcine Skeletal Muscle Tissue

**Figure 4:**
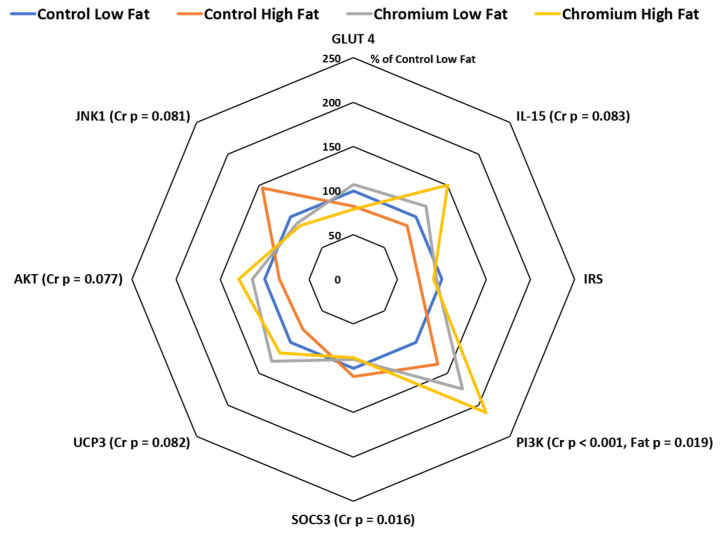
Interleukin-15 (IL-15), insulin receptor (IRS), phosphatidylinositol 3 kinase (PI3K), suppressor of cytokine signaling 3 (SOCS3), uncoupling protein 3 (UCP3), protein kinase B (AKT), mitogen-activated protein kinase-8 (JNK1) and glucose transporter 4 (GLUT4) mRNA expression in skeletal muscle tissue from gilts fed nCrPic with or without a high-fat diet. All gene expression data were analysed as the threshold cycle (cT) relative to that of *β*-actin (∆CT) and assessed for main and interactive effects of dietary fat and nCrPic by ANOVA. A difference in ∆CT of −1.0 is associated with a doubling (200%) and +1.0 a halving (50%) of expression and for ease of presentation data are presented as % relative to expression in tissue from gilts fed the control diet without supplemental fat or nCrPic. This method of presentation prevents the presentation of the error term.

The expression of IR, PI3K, AKT, GLUT4 and SOCS3 mRNA expression in porcine skeletal muscle tissue in response to dietary nCrPic and fat are shown in Figure 4. Dietary nCrPic up-regulated the expression of skeletal muscle tissue insulin signaling pathway gene PI3K (relative expression was 118 and 193% for main effect of control and nCrPic diets, respectively, *p* < 0.001) and AKT (relative expression was 92 and 122% for main effect of control and nCrPic diets, respectively, *p* = 0.08), but had no effect in IR (*p* = 0.71) and GLUT4 (*p* = 0.97) mRNA expression. The expression of SOCS3 in skeletal muscle tissue was lower in nCrPic pigs (relative expression was 105 and 89% for main effect of control and nCrPic diets, respectively, *p* = 0.02). 

### 3.6. UCP3, IL-15 and JNK1 Gene Expression in Porcine Skeletal Muscle Tissue

The expression of UCP3 mRNA tended to be higher in skeletal muscle in nCrPic animals (relative expression was 90 and 124% for main effect of control and nCrPic diets, respectively, *p* = 0.082). Similarly, the expression of interleukin-15 (IL-15) in skeletal muscle tissue tended to be higher in nCrPic animals (relative expression was 93 and 134% for main effect of control and nCrPic diets, respectively, *p* = 0.083). The expression of JNK1 tended to be decreased by dietary nCrPic (relative expression was 122 and 88% for main effect of control and nCrPic diets, respectively, *p* = 0.082) (Figure 4). A MANOVA of the changes in the expression of all eight genes in skeletal muscle indicated an overall significant effect of nCrPic (*p* = 0.042) and a trend for an effect of dietary fat (*p* = 0.094).

### 3.7. Correlations between mRNA Expression and Phenotypic Parameters

The correlation coefficients between the ∆CT for each gene and each other as well as phenotypic measures such as plasma HOMA and carcass back fat, loin muscle depth and carcass weight are provided as supplemental material (Table S3) because of complexity. As expected there were a number of significant correlations between the mRNA expression of related genes consistent with the effects of dietary nCrPic and fat. With respect to the phenotypic measures, there were a number of significant relationships with mRNA expression. For example, the ∆CT for adipose tissue ATK was positively associated with HOMA (*r* = 0.56, *p* = 0.024) and negatively associated with skeletal muscle JNK1 (r = −0.43, *p* < 0.10). (Table S3). A positive association means that the phenotypic parameter decreases as the gene mRNA expression increases and vice versa. Loin skeletal muscle depth was positively related to adipose tissue TNF*α* (*r* = 0.51, *p* = 0.043), PPAR*γ* (*r* = 0.53, *p* = 0.036) and GLUT4 (*r* = 0.58, *p* = 0.018) and negatively associated with adipose tissue FAS (*r* = −0.52, *p* = 0.040) and skeletal muscle IRS (*r* = −0.58, *p* = 0.019) and PI3K (*r* = −0.58, *p* = 0.020). Backfat depth was positively associated with adipose tissue PI3K (*r* = 0.67, *p* = 0.005) and AKT (*r* = 0.56, *p* = 0.026) and negatively associated with adipose tissue leptin (*r* = −0.51, *p* = 0.044) and SREBP (*r* = −0.56, *p* = 0.024) and skeletal muscle SOCS3 (*r* = −0.50, *p* = 0.046) and JNK1 (*r* = −0.57, *p* = 0.021).

## 4. Discussion

The essential trace mineral Cr has been suggested to have beneficial effects in individuals with type 2 diabetes as evidenced by enhance insulin sensitivity and glucose transport at the molecular level [4,28,29,30]. Dietary Cr can attenuate visceral fat accumulation and hence maintains healthy body composition in diabetic and overweight individuals [23,31]. However, the underlying mechanism by which Cr exerts its effects on the regulation of the insulin signaling pathway remains unclear. This study demonstrates that dietary nCrPic supplementation markedly increased PI3K and AKT expression in the muscle tissue and AKT in the subcutaneous adipose tissue indicating that dietary nCrPic can improve insulin sensitivity. These responses were associated with increased muscle mass and reduced subcutaneous fat accumulation. 

In muscle tissue, SOSC3 gene expression was down-regulated by dietary nCrPic supplementation. The members of SOCS family, especially SOCS3 have been implicated in cytokine-mediated inhibition of insulin signaling in adipose tissue, liver, and brain [32]. Furthermore, in skeletal muscle, SOCS3 is up-regulated after high-fat feeding or by IL-6 stimulation [33,34,35]. Over-expression of SOCS3 has also been shown to prevent leptin activation of AMPK signaling [34]. Therefore, elevated expression of SOCS3 in skeletal muscle may impair AMPK modulation of insulin-mediated glucose uptake [33,34]. Moreover, SOCS3 can bind to tyrosine-phosphorylated IRS1 and target IRS1 degradation via the SOCS box-mediated proteasomal complex [36]. In the present study, we found that both PI3K and AKT in muscle tissue were up-regulated by dietary nCrPic supplementation. These results indicated that the improvement of insulin sensitivity may be due to, at least in part, the reduction in SOCS3. 

The maintenance of plasma glucose homeostasis is a complex process involving responsiveness of several genes. The correct responses of IRS-PI3K-AKT are crucial for maintenance of insulin sensitivity. Serine phosphorylation of IRS1 can decrease IRS1 activity, due to conformational changes, causing IRS1 to be unable to associate with IR [37]. The most important role of IRS1 is to bind and activate PI3K. The gene PI3K is a key mediator of insulin signaling. The tyrosine phosphorylated IRS1 can activate PI3K through interacting with PI3K regulatory subunit p85. Activated PI3K generates the second messenger PIP3 which can activate AKT through phosphorylation. The activation of AKT facilitates translocation of GLUT4 to the sarcolemma to facilitates glucose uptake into the cell [38]. In the present study, skeletal muscle PI3K and AKT mRNA expression were up-regulated by nCrPic, indicating that nCrPic can improve insulin sensitivity. Cefalu et al. [39] demonstrated that supplementation of CrPic improves glucose disposal rates and enhances insulin-stimulated phosphorylation of IRS-1 and PI3K activity in skeletal muscle. The improvement of insulin signaling by Cr is associated with the decrease of IRS-1 Ser^307^ phosphorylation [25,40]. Further, serine phosphorylation of IRS-1 is a major target of JNK-mediated phosphorylation and elevated JNK phosphorylation occurs in insulin resistant cells in vitro. Elevated hepatic phospho-c-Jun has been observed in obese and insulin resistant mice [41], while tyrosine phosphorylation of c-Jun was suppressed by supplementation with chromium. Endoplasmic reticulum (ER) stress may play a role in the development of insulin resistance, and ER stress can activate JNK which in turn suppresses insulin signaling [42]. The results from the present experiment support these findings as JNK1 expression tended to be reduced in skeletal muscle of gilts fed nCrPic, while Özcan et al. [41] showed that inhibition of JNK activity reversed the ER-induced serine phosphorylation of IRS-1.

In the present study, UCP3 mRNA in skeletal muscle was increased by dietary nCrPic supplementation. In an in vitro study, Qiao et al. found that Cr improves glucose uptake through up-regulating the mRNA level of UCP3 in skeletal muscles [28]. UCP3 is another protein which is important for maintaining insulin sensitivity [43]. The increased expression of UCP3 is associated with increased carnitine palmitoyltransferase-1B, the enzyme associated with fatty acid oxidation [44,45]. Incomplete fatty acid oxidation can contribute to insulin resistance [46]. Gao et al. [47] also indicated that free fatty acid can activate c-JUN-NH2-Terminal Kinase and increase IRS-1 serine phosphorylation. Moreover, UCP3 also limits mitochondrial damage by suppression of lipid peroxidation and the production of reactive oxygen species [44]. IL-15, which is highly expressed in skeletal muscle tissue, has anabolic effects on skeletal muscle both in vivo and in vitro [48,49]. In adipocyte cultures, IL-15 also inhibited preadipocyte differentiation, and stimulated secretion of adiponectin from differentiated adipocytes [50]. In the present study, nCrPic also increased both IL-15 in skeletal muscle and adiponectin in subcutaneous adipose tissue. Additionally, administration of IL-15 resulted in an increasing of glucose uptake in both C2C12 cell culture and rat skeletal muscle [51]. Therefore, IL-15 could potentially stimulate adiponectin production by adipose tissue, which in turn may modulate skeletal muscle glucose metabolism and insulin sensitivity.

The effects of Cr on transcription factors are still unclear. Lien et al. [52] reported that 3T3-L1 preadipocytes cultured with 50 ppb CrPic exhibited decreased PPAR*γ* expression. However, there was no significant effect of dietary nCrPic supplementation on PPAR*γ* expression in the present study. Gene expression analysis in subcutaneous adipose tissue identified that dietary high-fat supplementation up-regulated both PPAR*γ* and C\EBP*α*. PPAR*γ* is highly expressed in adipocytes and is involved in adipocyte differentiation, lipid storage, and glucose homeostasis [53]. PPAR*γ* can be considered as the “master regulator” of adipogenesis, as no other factor can facilitate adipogenesis in the absence of PPAR*γ* [54]. The expression of C\EPBα occurs in the latter stages of the adipocyte differentiation process and has been shown to induce the differentiation of a variety of fibroblastic cells into adipocytes [55]. PPAR*γ* and C\EBP*α* cooperate to promote adipocyte differentiation, including adipocyte gene expression and insulin sensitivity [56]. Adipocytes also play a central role in maintaining energy balance. In addition, adipocytes produce and secrete adipokines which are involved in the insulin-signaling pathway [12,57]. In the present study, subcutaneous adipose tissue adiponectin mRNA expression was decreased by the high-fat diet and increased by dietary nCrPic, suggesting that nCrPic may able to reverse the high-fat-diet-induced defects in insulin signaling. Adiponectin is considered to be a marker of inflammation with lower concentrations exhibited in both obese humans and animal models of obesity/insulin resistance [58,59,60]. Several studies have shown a link between low circulating adiponectin concentrations and insulin resistance [61,62]. Jain et al. [63] reported that dietary Cr supplementation can lower blood glucose by elevating blood adiponectin in Zucker diabetic obese rats. In an obese *cp/cp* rat study, adiponectin level was not affected by CrPic alone, but was increased by a combination of CrPic and conjugated linoleic acid [64].

## 5. Conclusions

In conclusion, these data provide strong evidence that dietary nCrPic can improve insulin sensitivity in pigs consuming a high-fat diet. In particular, the expression of the insulin-signaling pathway genes PI3K and AKT were increased by dietary nCrPic. Furthermore, the expression of SOCS3 in skeletal muscle, which can aggravate insulin resistance, was reduced by nCrPic. Dietary nCrPic also increased UCP3 and IL-15 in skeletal muscle, both of which facilitate glucose metabolism. In subcutaneous adipose tissue, the expression of adiponectin was up-regulated by dietary nCrPic. These findings indicate the improvement in the insulin-signaling pathway by dietary nCrPic may be via decreased SOCS3 and increased UCP3 and IL-15 in skeletal muscle, as well as increased adiponectin in subcutaneous adipose tissue.

## Figures and Tables

**Table 1:** List of primers used for pig tissue gene expression.

Gene	Accession Number	Primer Sequence	Annealing Temp. (°C)	Amplicon Size (bp)	GC%
*β*-actin	DQ845171	For 5′ACATCCGCAAGGACCTCTAC3′ Rev 5′ACATCTGCTGGAAGGTGGAC3′	56.5 56.9	210	55 55
Insulin receptor	XM003123154.3	For 5′CAACACTGGTGGTGATGGAG3′ Rev 5′CCATCCCATCAGCAATCTCT3′	52.7 51.2	150	55 50
PI3K	NM213939	For 5′AACCTCCAGATCTACTGCGGCAAA3′ Rev5′AGGAAGCGGTGGTCTATCAGCAAT3′	60.1 60.0	134	50 50
AKT	NM001159776	For 5′TTCTACAACCAGGACCACGA3′ Rev 5′AATACCTGGTGTCCGTCTCG3′	52.4 53.4	268	50 50
GLUT4	NM001128433	For 5′GTCCAACTTCATCATCGGCA3′ Rev 5′ATGAAGAAGCCAAGCAGGAG3′	52.5 52.0	99	50 50
PPAR*γ*	AB097926	For 5′CTTTATGGAGCCCAAGTTCG3′ Rev 5′GAGGACTCTGGGTGGTTCAA3	50.8 53.0	200	50 55
C/EBP*α*	AF103944	For 5′GCTGACCAGTGACAATGACC3′ Rev 5′GGCACCGGAATCTCCTAGTC3′	55.4 58.8	250	55 60
SREBP-1	AY338729	For 5′TCCTTCCACCATGAGCTCCC3′ Rev 5′CACCGACGGGTACATCTTCA3′	55.3 53.7	118	60 55
JNK1	XM003359272.1	For 5′ACCTGACAAGCAGTTGGATG3′ Rev 5′TAGTCATCTACAGCAGCCCA3′	56.0 56.5	238	50 50
FAS	AY183428	For 5′TCGTGGGCTACAGCATGATA3′ Rev 5′GGAGTTAGGCTTCAGCAGGA3′	57.3 57.0	208	50 55
IL-15	NM214390	For 5′CAACCTGGCAGCACGTAAT3′ Rev5′CAGGAGAAAGCACTTCATCGCTGT3′	52.5 57.5	137	50 50
UCP3	NM214049	For 5′ACACAGATGTCCAGAGGTCA3′ Rev 5′CCAAACTCCACACCCTTCAA3′	58.0 57.9	182	50 50
SOCS3	AY944571	For 5′CTGGCTCTTTGATTTGGTTT3′ Rev 5′TGGACTCTGGGACCTGTATT3′	54.7 54.0	280	40 50
Adiponectin	EF601160	For 5′CTTGCGGGTCCTTGATAAAT3′ Rev 5′CCCCTAACCTCAGTGGAAAA3′	50.0 50.8	192	45 50
Leptin	NM213840	For 5′CCTCTGAATGGTCTGGGTTG3′ Rev 5′GGACTTGGGACCATCTGCTA3′	57.4 57.2	182	55 55
TNF-*α*	NM214022	For 5′CTGCCTTGGTTCAGATGTGT3′ Rev 5′CAGCGATGTAGCGACAAGTT3′	52.3 53.0	172	50 50

## Supplementary Materials

The following are available online at https://www.mdpi.com/2076-2615/10/9/1685/s1, Table S1: Effect of dietary nano CrPic and dietary fat on mitogen-activated protein kinase-8 (JNK1), leptin, adiponectin, tumor necrosis factor *α* (TNF*α*), peroxisome proliferator-activated receptor *γ* (PPAR*γ*), CCAAT enhancer binding protein *α* (C/EBP*α*), sterol regulatory element-binding protein (SREBP), fatty acid synthase (FAS), insulin receptor (IRS), phosphatidylinositol 3 kinase (PI3K), protein kinase B (AKT), uncoupling protein 3 (UCP3), suppressor of cytokine signaling 3 (SOCS3) and glucose transporter 4 (GLUT4) and mRNA expression in subcutaneous adipose tissue from gilts, Table S2: Effect of dietary nano CrPic and dietary fat on interleukin-15 (IL-15), insulin receptor (IRS), phosphatidylinositol 3 kinase (PI3K), suppressor of cytokine signaling 3 (SOCS3), uncoupling protein 3 (UCP3), protein kinase B (AKT), mitogen-activated protein kinase-8 (JNK1) and glucose transporter 4 (GLUT4) mRNA expression in skeletal muscle tissue from gilts, Table S3: Correlations (*r*) between mRNA expression (expressed as ∆CT) of adipose tissue mitogen-activated protein kinase-8 (JNK1), leptin, adiponectin, tumor necrosis factor *α* (TNF*α*), peroxisome proliferator-activated receptor *γ* (PPAR*γ*), CCAAT enhancer binding protein *α* (C/EBP*α*), sterol regulatory element-binding protein (SREBP), fatty acid synthase (FAS), insulin receptor (IRS), phosphatidylinositol 3 kinase (PI3K), protein kinase B (AKT), uncoupling protein 3 (UCP3), suppressor of cytokine signaling 3 (SOCS3) and glucose transporter 4 (GLUT4) and skeletal muscle interleukin-15 (IL-15), IRS, PI3K, SOCS3, UCP3, AKT, JNK1 and GLUT4 and homeostatic model assessment (HOMA), loin muscle depth, back fat depth and carcass weight in gilts.

## References

[1] Sell H., Dietze-Schroeder D., Eckel J. (2006). The adipocyte–myocyte axis in insulin resistance. Trends Endocrinol. Metab..

[2] Fryer L.G., Kruszynska Y.T. (1993). Insulin Resistance in High Fat Fed Rats. Ann. N. Y. Acad. Sci..

[3] Shearer J., Duggan G., Weljie A., Hittel D.S., Wasserman D.H., Vogel H.J. (2008). Metabolomic profiling of dietary-induced insulin resistance in the high fat-fed C57BL/6J mouse. Diabetes Obes. Metab..

[4] Kandadi M.R., Unnikrishnan M.K., Warrier A.K.S., Du M., Ren J., Nair S. (2011). Chromium (d-Phenylalanine)3 alleviates high fat-induced insulin resistance and lipid abnormalities. J. Inorg. Biochem..

[5] Qatanani M., Lazar M.A. (2007). Mechanisms of obesity-associated insulin resistance: Many choices on the menu. Genes Dev..

[6] Dunshea F.R., Cox M.L. (2008). Effect of dietary protein on body composition and insulin resistance using a pig model of the child and adolescent. Nutr. Diet..

[7] Sabin M., Yau S.W., Russo V.C., Clarke I.J., Dunshea F.R., Chau J., Cox M., Werther G. (2011). Dietary Monounsaturated Fat in Early Life Regulates IGFBP2: Implications for Fat Mass Accretion and Insulin Sensitivity. Obesity.

[8] Dunshea F.R., D’Souza D.N., Paterson J.E. (2003). Review: Fat deposition and metabolism in the pig. Manipulating Pig Production IX.

[9] Cao Z., Umek R.M., McKnight S.L. (1991). Regulated expression of three C/EBP isoforms during adipose conversion of 3T3-L1 cells. Genes Dev..

[10] Rosen E.D., Spiegelman B.M. (2000). Molecular Regulation of Adipogenesis. Annu. Rev. Cell Dev. Biol..

[11] Horton J.D., Shimomura I., Brown M.S., Hammer R., Goldstein J.L., Shimano H. (1998). Activation of cholesterol synthesis in preference to fatty acid synthesis in liver and adipose tissue of transgenic mice overproducing sterol regulatory element-binding protein-2. J. Clin. Investig..

[12] Galic S., Oakhill J.S., Steinberg G.R. (2010). Adipose tissue as an endocrine organ. Mol. Cell. Endocrinol..

[13] Hotamisligil G.S., Arner P., Caro J.F., Atkinson R.L., Spiegelman B.M. (1995). Increased adipose tissue expression of tumor necrosis factor-alpha in human obesity and insulin resistance. J. Clin. Investig..

[14] Lihn A.S., Pedersen S.B., Richelsen B. (2005). Adiponectin: Action, regulation and association to insulin sensitivity. Obes. Rev..

[15] Kamohara S., Burcelin R., Halaas J.L., Friedman J.M., Charron M.J. (1997). Acute stimulation of glucose metabolism in mice by leptin treatment. Nature.

[16] Ogawa Y., Masuzaki H., Hosoda K., Aizawa-Abe M., Suga J., Suda M., Ebihara K., Iwai H., Matsuoka N., Satoh N. (1999). Increased glucose metabolism and insulin sensitivity in transgenic skinny mice overexpressing leptin. Diabetes.

[17] Steinberg G.R., Dyck D.J. (2000). Development of leptin resistance in rat soleus muscle in response to high-fat diets. Am. J. Physiol. Metab..

[18] Mertz W., Roginski E.E. (1969). Effects of Chromium(III) Supplementation on Growth and Survival Under Stress in Rats Fed Low Protein Diets. J. Nutr..

[19] Mertz W. (1993). Chromium in Human Nutrition: A Review. J. Nutr..

[20] Amoikon E.K., Fernandez J.M., Southern L.L., Thompson D.L., Ward T.L., Olcott B.M. (1995). Effect of chromium tripicolinate on growth, glucose tolerance, insulin sensitivity, plasma metabolites, and growth hormone in pigs2. J. Anim. Sci..

[21] Jeejeebhoy K.N., Chu R.C., Marliss E.B., Greenberg G.R., Bruce-Robertson A. (1977). Chromium deficiency, glucose intolerance, and neuropathy reversed by chromium supplementation, in a patient receiving long-term total parenteral nutrition. Am. J. Clin. Nutr..

[22] Padmavathi I.J., Rao K.R., Venu L., Ganeshan M., Kumar K.A., Rao C.N., Harishankar N., Ismail A., Raghunath M. (2009). Chronic Maternal Dietary Chromium Restriction Modulates Visceral Adiposity: Probable Underlying Mechanisms. Diabetes.

[23] Martin J., Wang Z.Q., Zhang X.H., Wachtel D., Volaufova J., Matthews D.E., Cefalu W.T. (2006). Chromium Picolinate Supplementation Attenuates Body Weight Gain and Increases Insulin Sensitivity in Subjects With Type 2 Diabetes. Diabetes Care.

[24] Chen T.J.H., Blum K., Kaats G., Braverman E.R., Eisenberg A., Sherman M., Davis K., Comings D.E., Wood R., Pullin D. (2007). Chromium Picolinate (CrP) a putative anti-obesity nutrient induces changes in body composition as a function of the Taq1 dopamine D2 receptor polymorphisms in a randomized double-blind placebo controlled study. Gene. Ther. Mol. Biol..

[25] Chen W.-Y., Chen C.-J., Liu C.-H., Mao F.C. (2009). Chromium supplementation enhances insulin signalling in skeletal muscle of obese KK/HlJ diabetic mice. Diabetes Obes. Metab..

[26] Hung A.T., Leury B.J., Sabin M., Lien T.F., Dunshea F.R. (2015). Dietary chromium picolinate of varying particle size improves carcass characteristics and insulin sensitivity in finishing pigs fed low- and high-fat diets. Anim. Prod. Sci..

[27] Katz A., Nambi S.S., Mather K.J., Baron A.D., Follmann D.A., Sullivan G., Quon M.J. (2000). Quantitative Insulin Sensitivity Check Index: A Simple, Accurate Method for Assessing Insulin Sensitivity In Humans. J. Clin. Endocrinol. Metab..

[28] Qiao W., Peng Z., Wang Z., Wei J., Zhou A. (2009). Chromium Improves Glucose Uptake and Metabolism Through Upregulating the mRNA Levels of IR, GLUT4, GS, and UCP3 in Skeletal Muscle Cells. Biol. Trace Elem. Res..

[29] Wang Z.Q., Cefalu W.T. (2010). Current Concepts about Chromium Supplementation in Type 2 Diabetes and Insulin Resistance. Curr. Diabetes Rep..

[30] Yan X., Zhang F., Li N., Zhu X., Jia Z. (2009). Effects of Chromium on Energy Metabolism in Lambs Fed with Different Dietary Protein Levels. Asian-Australas. J. Anim. Sci..

[31] Crawford V., Scheckenbach R., Preuss H.G. (1999). Effects of niacin-bound chromium supplementation on body composition in overweight African-American women. Diabetes Obes. Metab..

[32] Howard J.K., Flier J.S. (2006). Attenuation of leptin and insulin signaling by SOCS proteins. Trends Endocrinol. Metab..

[33] Rieusset J., Bouzakri K., Chevillotte E., Ricard N., Jacquet D., Bastard J.-P., Laville M., Vidal H. (2004). Suppressor of cytokine signaling 3 expression and insulin resistance in skeletal muscle of obese and type 2 diabetic patients. Diabetes.

[34] Steinberg G.R., McAinch A., Chen M.B., O’Brien P.E., Dixon J.B., Cameron-Smith D., Kemp B. (2006). The Suppressor of Cytokine Signaling 3 Inhibits Leptin Activation of AMP-Kinase in Cultured Skeletal Muscle of Obese Humans. J. Clin. Endocrinol. Metab..

[35] Watt M.J., Dzamko N., Thomas W.G., Rose-John S., Ernst M., Carling D., Kemp B., Febbraio M., Steinberg G.R. (2006). CNTF reverses obesity-induced insulin resistance by activating skeletal muscle AMPK. Nat. Med..

[36] Rui L., Yuan M., Frantz D., Shoelson S., White M.F. (2002). SOCS-1 and SOCS-3 Block Insulin Signaling by Ubiquitin-mediated Degradation of IRS1 and IRS2. J. Biol. Chem..

[37] Virkamäki A., Ueki K., Kahn C.R. (1999). Protein–protein interaction in insulin signaling and the molecular mechanisms of insulin resistance. J. Clin. Investig..

[38] Saltiel A.R., Kahn C.R. (2001). Insulin signalling and the regulation of glucose and lipid metabolism. Nature.

[39] Cefalu W.T., Wang Z.Q., Zhang X.H., Baldor L.C., Russell J.C. (2002). Oral chromium picolinate improves carbohydrate and lipid metabolism and enhances skeletal muscle Glut-4 translocation in obese, hyperinsulinemic (JCR-LA corpulent) rats. J. Nutr..

[40] Sreejayan N., Dong F., Kandadi M.R., Yang X., Ren J. (2008). Chromium Alleviates Glucose Intolerance, Insulin Resistance, and Hepatic ER Stress in Obese Mice. Obesity.

[41] Hua Y., Clark S., Ren J., Nair S. (2012). Molecular mechanisms of chromium in alleviating insulin resistance. J. Nutr. Biochem..

[42] Ozcan U. (2004). Endoplasmic Reticulum Stress Links Obesity, Insulin Action, and Type 2 Diabetes. Science.

[43] Huppertz C., Fischer B.M., Kim Y.-B., Kotani K., Vidal-Puig A., Slieker L.J., Sloop K.W., Lowell B.B., Kahn B.B. (2001). Uncoupling Protein 3 (UCP3) Stimulates Glucose Uptake in Muscle Cells through a Phosphoinositide 3-Kinase-dependent Mechanism. J. Biol. Chem..

[44] MacLellan J.D., Gerrits M.F., Gowing A., Smith P.J.S., Wheeler M.B., Harper M.-E. (2005). Physiological Increases in Uncoupling Protein 3 Augment Fatty Acid Oxidation and Decrease Reactive Oxygen Species Production Without Uncoupling Respiration in Muscle Cells. Diabetes.

[45] Chan C.B., Harper M.-E. (2006). Uncoupling proteins: Role in insulin resistance and insulin insufficiency. Curr. Diabetes Rev..

[46] Zhang L., Keung W., Samokhvalov V., Wang W., Lopaschuk G.D. (2010). Role of fatty acid uptake and fatty acid β-oxidation in mediating insulin resistance in heart and skeletal muscle. Biochim. Biophys. Acta (BBA) Mol. Cell Biol. Lipids.

[47] Gao Z., Zhang X., Zuberi A., Hwang D., Quon M.J., Lefevre M., Ye J. (2004). Inhibition of Insulin Sensitivity by Free Fatty Acids Requires Activation of Multiple Serine Kinases in 3T3-L1 Adipocytes. Mol. Endocrinol..

[48] Quinn L.S., Anderson B.G., Drivdahl R.H., Alvarez B., Argilés J.M. (2002). Overexpression of interleukin-15 induces skeletal muscle hypertrophy in vitro: Implications for treatment of muscle wasting disorders. Exp. Cell Res..

[49] Busquets S., Figueras M.T., Meijsing S., Carbó N., Quinn L.S., Almendro V., Argilés J.M., López-Soriano F.J. (2005). Interleukin-15 decreases proteolysis in skeletal muscle: A direct effect. Int. J. Mol. Med..

[50] Quinn L.S., Strait-Bodey L., Anderson B.G., Argilés J.M., Havel P. (2005). Interleukin-15 stimulates adiponectin secretion by 3T3-L1 adipocytes: Evidence for a skeletal muscle-to-fat signaling pathway. Cell Biol. Int..

[51] Busquets S., Figueras M., Almendro V., López-Soriano F.J., Argilés J.M. (2006). Interleukin-15 increases glucose uptake in skeletal muscle an antidiabetogenic effect of the cytokine. Biochim. Biophys. Acta (BBA) Gen. Subj..

[52] Lien T.-F., Wu C.-P., Horng Y.-M. (2007). Chromium picolinate depressed proliferation and differentiation of 3T3-L1 preadipocytes. Nutr. Res..

[53] Lee C.-H., Olson P., Evans R.M. (2003). Minireview: Lipid Metabolism, Metabolic Diseases, and Peroxisome Proliferator-Activated Receptors. Endocrinology.

[54] Rosen E.D., MacDougald O.A. (2006). Adipocyte differentiation from the inside out. Nat. Rev. Mol. Cell Biol..

[55] Freytag S., Paielli D.L., Gilbert J.D. (1994). Ectopic expression of the CCAAT/enhancer-binding protein alpha promotes the adipogenic program in a variety of mouse fibroblastic cells. Genes Dev..

[56] Wu Z., Rosen E.D., Brun R., Hauser S., Adelmant G., Troy A., McKeon C., Darlington G.J., Spiegelman B.M. (1999). Cross-Regulation of C/EBPα and PPARγ Controls the Transcriptional Pathway of Adipogenesis and Insulin Sensitivity. Mol. Cell.

[57] Ahima R.S., Lazar M.A. (2008). Adipokines and the peripheral and neural control of energy balance. Mol. Endocrinol..

[58] Kubota N., Terauchi Y., Yamauchi T., Kubota T., Moroi M., Matsui J., Eto K., Yamashita T., Kamon J., Satoh H. (2002). Disruption of Adiponectin Causes Insulin Resistance and Neointimal Formation. J. Biol. Chem..

[59] Díez J.J., Iglesias P. (2003). The role of the novel adipocyte-derived hormone adiponectin in human disease. Eur. J. Endocrinol..

[60] Kadowaki T., Yamauchi T., Kubota N., Hara K., Ueki K., Tobe K. (2006). Adiponectin and adiponectin receptors in insulin resistance, diabetes, and the metabolic syndrome. J. Clin. Investig..

[61] Swarbrick M.M., Havel P. (2008). Physiological, Pharmacological, and Nutritional Regulation of Circulating Adiponectin Concentrations in Humans. Metab. Syndr. Relat. Disord..

[62] Yaturu S., Daberry R.P., Rains J., Jain S.K. (2006). Resistin and adiponectin levels in subjects with coronary artery disease and type 2 diabetes. Cytokine.

[63] Jain S.K., Croad J.L., Velusamy T., Rains J.L., Bull R. (2010). Chromium dinicocysteinate supplementation can lower blood glucose, CRP, MCP-1, ICAM-1, creatinine, apparently mediated by elevated blood vitamin C and adiponectin and inhibition of NFκB, Akt, and Glut-2 in livers of zucker diabetic fatty rats. Mol. Nutr. Food Res..

[64] Proctor S.D., Kelly S.E., Stanhope K.L., Havel P., Russell J.C. (2007). Synergistic effects of conjugated linoleic acid and chromium picolinate improve vascular function and renal pathophysiology in the insulin-resistant JCR:LA-cp rat. Diabetes Obes. Metab..

