# Supplementary material for: Nano Chromium Picolinate Improves Gene Expression Associated with Insulin Signaling in Porcine Skeletal Muscle and Adipose Tissue
Source: Animals (Basel). 2020 Sep 18;10(9):1685. doi: 10.3390/ani10091685 (PMC7552722; doi:10.3390/ani10091685)
Supplement: Supplementary file 1 [file animals-10-01685-s001.pdf]

**Table S1.** Effect of dietary nano CrPic and dietary fat on mitogen-activated protein kinase-8 (JNK1), leptin, adiponectin, tumor necrosis factor *α* (TNF*α*), peroxisome proliferator-activated receptor *γ* (PPAR*γ*), CCAAT enhancer binding protein *α* (C/EBP*α*), sterol regulatory element-binding protein (SREBP), fatty acid synthase (FAS), insulin receptor (IRS), phosphatidylinositol 3 kinase (PI3K), protein kinase B (AKT), uncoupling protein 3 (UCP3), suppressor of cytokine signalling 3 (SOCS3) and glucose transporter 4 (GLUT4) and mRNA expression in subcutaneous adipose tissue from gilts. Data are expressed as fed with nCrPic with or without a high fat diet. All gene expression data were analysed as the threshold cycle (cT) relative to that of the housekeeping gene *β*-actin (ΔCT) and assessed for main and interactive effects of dietary fat and nCrPic by ANOVA. Data are means and standard error of the difference for the interaction between dietary nCrPic and fat.

| nCrPic (Cr), ppb | 0 | | 400 | | | p-value | | |
|---|---|---|---|---|---|---|---|---|
| Fat (g/kg) | 22 | 57 | 22 | 57 | sed | Cr | Fat | Cr x Fat |
| JNK1 | 7.09 | 6.11 | 7.01 | 6.48 | 0.423 | 0.64 | 0.032 | 0.48 |
| Leptin | 7.72 | 5.25 | 6.04 | 6.01 | 0.717 | 0.39 | 0.036 | 0.040 |
| Adiponection | 15.1 | 15.8 | 15.0 | 15.2 | 0.24 | 0.067 | 0.016 | 0.21 |
| TNF*α* | 9.78 | 9.04 | 10.2 | 9.58 | 0.541 | 0.23 | 0.10 | 0.90 |
| PPAR*γ* | 5.35 | 4.28 | 5.70 | 4.77 | 0.648 | 0.38 | 0.058 | 0.88 |
| C/EBP*α* | 4.88 | 3.01 | 4.10 | 3.09 | 0.695 | 0.49 | 0.017 | 0.40 |
| SREBP | 14.7 | 15.3 | 14.5 | 15.9 | 1.07 | 0.79 | 0.22 | 0.62 |
| FAS | 13.5 | 14.3 | 12.6 | 13.5 | 6.360 | 0.85 | 0.86 | 1.00 |
| IRS | 7.49 | 7.59 | 7.58 | 7.37 | 0.76 | 0.91 | 0.93 | 0.78 |
| PI3K | 15.2 | 15.1 | 15.0 | 13.9 | 1.15 | 0.41 | 0.47 | 0.58 |
| AKT | 4.77 | 4.69 | 4.03 | 3.73 | 0.452 | 0.026 | 0.56 | 0.75 |
| UCP3 | 5.86 | 4.36 | 3.92 | 3.60 | 0.464 | 0.003 | 0.021 | 0.10 |
| SOCS3 | 6.09 | 5.87 | 5.84 | 5.57 | 0.707 | 0.60 | 0.64 | 0.96 |
| GLUT4 | 13.3 | 12.1 | 12.0 | 13.8 | 1.41 | 0.83 | 0.76 | 0.17 |

**Table S2.** Effect of dietary nano CrPic and dietary fat on interleukin-15 (IL-15), insulin receptor (IRS), phosphatidylinositol 3 kinase (PI3K), suppressor of cytokine signaling 3 (SOCS3), uncoupling protein 3 (UCP3), protein kinase B (AKT), mitogen-activated protein kinase-8 (JNK1) and glucose transporter 4 (GLUT4) mRNA expression in skeletal muscle tissue from gilts. Data are expressed as fed with nCrPic with or without a high fat diet. All gene expression data were analysed as the threshold cycle (cT) relative to that of the housekeeping gene *β*-actin (ΔCT) and assessed for main and interactive effects of dietary fat and nCrPic by ANOVA. Data are means and standard error of the difference for the interaction between dietary nCrPic and fat.

| nCrPic (Cr), ppb | 0 | | 400 | | | p-value | | |
|---|---|---|---|---|---|---|---|---|
| Fat (g/kg) | 22 | 57 | 22 | 57 | sed | Cr | Fat | Cr x Fat |
| IL-15 | 8.07 | 8.29 | 7.85 | 7.48 | 0.385 | 0.083 | 0.78 | 0.30 |
| IRS | 5.59 | 6.02 | 5.68 | 5.73 | 0.369 | 0.71 | 0.38 | 0.47 |
| PI3K | 9.91 | 9.47 | 9.10 | 8.82 | 0.177 | <.001 | 0.019 | 0.56 |
| SOCS3 | 6.32 | 6.19 | 6.47 | 6.51 | 0.110 | 0.016 | 0.54 | 0.32 |
| UCP3 | 3.07 | 3.39 | 2.69 | 2.84 | 0.348 | 0.082 | 0.35 | 0.74 |
| AKT | 4.99 | 5.25 | 4.80 | 4.62 | 0.299 | 0.077 | 0.85 | 0.33 |
| JNK1 | 5.99 | 5.45 | 6.15 | 6.22 | 0.346 | 0.081 | 0.36 | 0.24 |
| GLUT4 | 2.74 | 3.02 | 2.64 | 3.08 | 0.521 | 0.97 | 0.35 | 0.83 |

**Table S3.** Correlations (*r*) between mRNA expression (expressed as ΔCT) of adipose tissue mitogen-activated protein kinase-8 (JNK1), leptin, adiponectin, tumor necrosis factor $\alpha$ (TNF$\alpha$), peroxisome proliferator-activated receptor $\gamma$ (PPAR$\gamma$), CCAAT enhancer binding protein $\alpha$ (C/EBP$\alpha$), sterol regulatory element-binding protein (SREBP), fatty acid synthase (FAS), insulin receptor (IRS), phosphatidylinositol 3 kinase (PI3K), protein kinase B (AKT), uncoupling protein 3 (UCP3), suppressor of cytokine signalling 3 (SOCS3) and glucose transporter 4 (GLUT4) and skeletal muscle interleukin-15 (IL-15), IRS, PI3K, SOCS3, UCP3, AKT, JNK1 and GLUT4 and homeostatic model assessment (HOMA), loin muscle depth, back fat depth and carcass weight in gilts. For $|r| >$ 0.41, 0.48, 0.61 and 0.72 then $p < 0.10$, 0.05, 0.01 and 0.001, respectively.

| | | | | | | | | | | | | | | |
|---|---|---|---|---|---|---|---|---|---|---|---|---|---|---|
| Adipose JNK1 | 1 | - | | | | | | | | | | | | |
| Adipose Leptin | 2 | 0.33 | - | | | | | | | | | | | |
| Adipose Adiponection | 3 | -0.11 | -0.50 | - | | | | | | | | | | |
| Adipose TNF$\alpha$ | 4 | 0.50 | 0.25 | -0.40 | - | | | | | | | | | |
| Adipose PPAR$\gamma$ | 5 | 0.48 | 0.09 | 0.16 | 0.36 | - | | | | | | | | |
| Adipose C/EBP$\alpha$ | 6 | 0.29 | 0.69 | -0.32 | -0.12 | 0.14 | - | | | | | | | |
| Adipose SREBP | 7 | 0.26 | 0.27 | 0.15 | 0.07 | -0.18 | -0.06 | - | | | | | | |
| Adipose FAS | 8 | -0.23 | 0.11 | -0.21 | -0.48 | -0.29 | 0.29 | -0.02 | - | | | | | |
| Adipose IRS | 9 | 0.35 | -0.14 | 0.42 | 0.00 | 0.56 | 0.16 | 0.05 | -0.33 | - | | | | |
| Adipose PI3K | 10 | 0.23 | -0.16 | 0.24 | 0.49 | 0.37 | -0.34 | -0.26 | -0.82 | 0.31 | - | | | |
| Adipose AKT | 11 | 0.35 | 0.05 | 0.31 | 0.07 | 0.35 | 0.02 | 0.01 | -0.18 | 0.41 | 0.49 | - | | |
| Adipose UCP3 | 12 | 0.59 | 0.33 | 0.20 | 0.01 | 0.12 | 0.41 | 0.00 | -0.14 | 0.34 | 0.33 | 0.56 | - | |
| Adipose SOCS3 | 13 | 0.39 | 0.39 | 0.17 | 0.28 | 0.21 | 0.21 | 0.48 | -0.44 | 0.50 | 0.42 | 0.34 | 0.45 | - |
| Adipose GLUT4 | 14 | 0.12 | -0.08 | 0.08 | 0.40 | -0.06 | -0.25 | 0.06 | -0.40 | 0.10 | 0.40 | 0.05 | 0.20 | 0.19 |
| Muscle IL15 | 15 | 0.03 | -0.23 | 0.38 | -0.47 | -0.07 | 0.10 | -0.04 | -0.14 | 0.39 | 0.22 | 0.53 | 0.43 | 0.31 |
| Muscle IRS | 16 | -0.26 | -0.12 | -0.05 | -0.10 | -0.64 | -0.16 | 0.33 | 0.27 | -0.45 | -0.49 | -0.47 | -0.21 | -0.25 |
| Muscle PI3K | 17 | -0.07 | 0.49 | -0.36 | -0.07 | -0.49 | 0.41 | 0.15 | 0.23 | -0.36 | -0.30 | 0.06 | 0.27 | 0.10 |
| Muscle SOCS3 | 18 | 0.28 | 0.10 | 0.16 | -0.25 | 0.22 | 0.30 | 0.38 | 0.33 | 0.02 | -0.58 | -0.33 | -0.10 | -0.10 |
| Muscle UCP3 | 19 | -0.18 | 0.08 | 0.47 | -0.64 | -0.19 | 0.14 | 0.27 | 0.05 | 0.07 | -0.06 | 0.29 | 0.23 | 0.29 |
| Muscle AKT | 20 | 0.15 | -0.22 | 0.50 | -0.26 | 0.01 | -0.18 | 0.17 | -0.34 | 0.35 | 0.44 | 0.64 | 0.43 | 0.47 |
| Muscle JNK1 | 21 | 0.41 | 0.45 | -0.44 | 0.63 | 0.18 | 0.10 | 0.36 | 0.02 | -0.25 | -0.23 | -0.40 | -0.18 | -0.03 |
| Muscle GLUT4 | 22 | -0.49 | -0.29 | 0.22 | -0.62 | -0.38 | -0.08 | -0.02 | -0.04 | -0.10 | -0.29 | -0.41 | -0.23 | -0.28 |
| HOMA | 23 | -0.22 | 0.16 | 0.36 | -0.15 | 0.12 | 0.18 | -0.17 | -0.23 | 0.22 | 0.39 | 0.56 | 0.34 | 0.25 |
| Muscle depth | 24 | 0.33 | -0.09 | -0.01 | 0.51 | 0.53 | -0.11 | -0.11 | -0.52 | 0.34 | 0.48 | 0.06 | -0.02 | 0.15 |
| Fat depth | 25 | 0.05 | -0.51 | 0.21 | 0.12 | 0.30 | -0.28 | -0.56 | -0.42 | 0.35 | 0.67 | 0.56 | 0.20 | -0.04 |
| Carcass weight | 26 | -0.03 | -0.48 | 0.25 | 0.09 | 0.44 | -0.30 | -0.54 | -0.49 | 0.32 | 0.57 | 0.22 | -0.05 | -0.18 |
| | | 1 | 2 | 3 | 4 | 5 | 6 | 7 | 8 | 9 | 10 | 11 | 12 | 13 |

| | | | | | | | | | | | | | | |
|---|---|---|---|---|---|---|---|---|---|---|---|---|---|---|
| Adipose GLUT4 | 14 | - | | | | | | | | | | | | |
| Muscle IL15 | 15 | -0.09 | - | | | | | | | | | | | |
| Muscle IRS | 16 | -0.12 | -0.18 | - | | | | | | | | | | |
| Muscle PI3K | 17 | -0.15 | 0.20 | 0.52 | - | | | | | | | | | |
| Muscle SOCS3 | 18 | -0.37 | -0.15 | 0.14 | -0.19 | - | | | | | | | | |
| Muscle UCP3 | 19 | -0.11 | 0.62 | -0.18 | 0.16 | 0.08 | - | | | | | | | |
| Muscle AKT | 20 | 0.05 | 0.89 | -0.22 | 0.08 | -0.22 | 0.66 | - | | | | | | |
| Muscle JNK1 | 21 | 0.05 | -0.83 | 0.26 | 0.01 | 0.36 | -0.58 | -0.69 | - | | | | | |
| Muscle GLUT4 | 22 | -0.15 | 0.35 | 0.39 | 0.13 | 0.21 | 0.29 | 0.16 | -0.37 | - | | | | |
| HOMA | 23 | -0.01 | 0.27 | -0.36 | 0.12 | -0.32 | 0.29 | 0.25 | -0.43 | -0.03 | - | | | |
| Muscle depth | 24 | 0.58 | -0.19 | -0.58 | -0.58 | 0.01 | -0.34 | -0.11 | 0.18 | -0.25 | 0.06 | - | | |
| Fat depth | 25 | 0.28 | 0.48 | -0.48 | -0.23 | -0.50 | -0.07 | 0.46 | -0.57 | -0.08 | 0.34 | 0.45 | - | |
| Carcass weight | 26 | 0.36 | 0.13 | -0.63 | -0.60 | -0.23 | -0.11 | 0.13 | -0.34 | 0.03 | 0.28 | 0.74 | 0.80 | - |
| | | 14 | 15 | 16 | 17 | 18 | 19 | 20 | 21 | 22 | 23 | 24 | 25 | 26 |
